# LGMN^+^ macrophage promotes the formation of a tumor-supportive microenvironment in gastric cancer

**DOI:** 10.3389/fimmu.2026.1810794

**Published:** 2026-04-14

**Authors:** Weixin Zhang, Dapeng Chen, Jinming Li, Tianze Wang, Chengyu Jia, Yi Bai, Yamin Zhang

**Affiliations:** 1 First Central Hospital of Tianjin Medical University, Tianjin Medical University, Tianjin, China; 2 School of Medicine, Nankai University, Tianjin, China; 3 Department of Hepatobiliary Surgery, Tianjin First Central Hospital, Tianjin, China

**Keywords:** angiogenesis, gastric cancer, immunosuppression, legumain, macrophage

## Abstract

**Background:**

Tumor-associated macrophages (TAMs) are known to facilitate cancer progression. However, the diversity of TAM subsets and their distinct roles in GC remain poorly understood. This study aimed to evaluate the impact of legumain (LGMN)^+^ macrophages on GC progression and clarify the underlying mechanisms of their roles.

**Methods:**

We used single-cell RNA sequencing (scRNA-seq) and bulk RNA sequencing (bulk RNA-seq) analyses from public databases (GEO and TCGA) to systematically evaluate the clinical prognostic significance of LGMN and to characterize the remodeling of its associated signaling pathways. To investigate the role of LGMN in mouse GC (TAMs), we generated macrophage-specific LGMN conditional knockout mice, LGMN^flox/flox^; *Lyz2-Cre*. Utilizing a combination of subcutaneous xenograft tumor models, primary cell isolation and culture, immunofluorescence staining, and tube formation assays, we systematically elucidated the regulatory function and underlying molecular mechanisms of LGMN^+^ macrophages in GC progression.

**Results:**

We found that LGMN^+^ macrophages are significantly enriched in GC tissues, and their high infiltration was significantly associated with poor outcomes. Additionally, scRNA-seq revealed that hypoxia and immune suppression pathways are enriched in LGMN^+^ macrophages. LGMN^+^ macrophages infiltration levels showed a significant positive correlation with the infiltration of regulatory T (Treg) cells and endothelial cells. Mechanistically, conditional knockout of LGMN in macrophages inhibits tumor growth by reprogramming TAMs toward an anti-tumor phenotype, reducing Treg cell infiltration, and enhancing the infiltration level of CD8^+^ T cells. Furthermore, LGMN knockout can inhibit tumor angiogenesis by downregulating VEGF-A expression.

**Conclusions:**

LGMN^+^ macrophages drive GC progression by promoting tumor angiogenesis and establishing an immunosuppressive microenvironment. Therefore, targeting this TAM subset may represent a novel therapeutic strategy for GC.

## Introduction

Gastric cancer (GC) is among the most common malignancies worldwide (1), ranking fifth globally in terms of global incidence and cancer-related mortality, with the highest incidence rates observed in Eastern Asia (2). The 5-year survival rate post-surgery is 60%–80% for patients with stage IA and IB disease; however, it drops markedly to 18%–50% for those with stage III tumors (3). Chemotherapy, using agents such as fluorouracil (5-FU) or capecitabine, is the standard of care for patients with advanced GC. However, the clinical efficacy of these conventional regimens is often limited. The median overall survival (mOS) for patients with advanced GC receiving such therapy is approximately 8 months (4). The emergence of cancer immunotherapy has resulted in transformative advances in the clinical treatment of various cancer types (5). An increasing number of immunotherapy regimens are currently in clinical use or under active investigation (4). Immunotherapy has attained notable progress, but some important challenges remain. However, the efficacy of monotherapy is typically low, and the population that benefits from it remains limited. Recent clinical trials have demonstrated that the PD-1 inhibitor pembrolizumab, used as monotherapy and in combination with chemotherapy, is not inferior to chemotherapy alone in advanced GC; however, this combination did not yield a statistically significant improvement in overall survival (OS) or progression-free survival (PFS) (6). A phase II trial assessed the cytotoxic T-lymphocyte-associated protein 4 (CTLA-4) inhibitor ipilimumab in patients with advanced/metastatic gastric or gastroesophageal junction cancer who had attained at least stable disease following first-line chemotherapy. Although a 10 mg/kg dose demonstrated an acceptable safety profile, it did not markedly enhance immune-related progression-free survival compared to best supportive care (7). Similarly, maintenance avelumab did not demonstrate superiority over chemotherapy in OS for patients with advanced GC after first-line induction chemotherapy (8). Therefore, understanding the mechanisms that restrict the efficacy of immunotherapy is essential for improving its clinical benefit.

The progression of GC is facilitated by its complex tumor microenvironment (TME), which enables immune evasion. Tumor-associated macrophages (TAMs) are among the most abundant immune cells within the TME. TAMs differentiate in response to various cytokines and growth factors, typically classified into M1 and M2 subtypes. M2 macrophages are stimulated by interleukin-4 (IL-4), interleukin-10 (IL-10), interleukin-13 (IL-13), and glucocorticoids, displaying anti-inflammatory properties. They facilitate tumor growth and metastasis by enhancing immunosuppression, angiogenesis, and resistance to drug therapy, and they are typically associated with poor prognosis. Targeting M2 macrophages infiltration and proliferation constitutes a highly promising approach for cancer immunotherapy. Therefore, it is essential to identify the core regulatory molecules that can regulate the immunosuppressive functions of M2 macrophages. Legumain (LGMN) is a cysteine endopeptidase classified within the C13 peptidase family, which specifically hydrolyzes substrate proteins at asparaginyl bonds (9, 10). LGMN is overexpressed in various cancers, including glioblastoma, GC, breast cancer, and cervical cancer. Previous studies have reported that LGMN facilitates cancer cell invasion and metastasis. Nevertheless, it is associated with tumor immune suppression. Pang et al. reported that inhibiting LGMN reduces macrophage M2 polarization, amplifies CD8^+^ T cell-mediated anti-tumor immunity, and synergizes with anti-PD1 therapy in a glioblastoma mouse model (11). The functions of LGMN in GC remains unclear. Recent findings demonstrate that GC cells secrete LGMN, which facilitates M2 polarization of macrophages and suppresses anti-tumor immunity (12). Subsequent studies by Wang et al. demonstrated that LGMN is primarily expressed in macrophages and facilitates angiogenesis (13). Therefore, it is unclear whether LGMN^+^ macrophages represent a unique, functionally specialized subset within the GC TME and how they systematically modulate immunity and angiogenesis.

This study utilized single-cell sequencing and macrophage-specific LGMN-knockout mice to examine the role of LGMN in GC. Our analysis successfully identified a macrophage subpopulation highly expressing LGMN, which suppressed the anti-tumor immunity. We observed that patients with GC with higher infiltration of LGMN^+^ macrophages exhibited a significantly worse prognosis. The frequency of LGMN^+^ macrophages exhibits a strong association with regulatory T (Treg) cells, facilitating the recruitment of regulatory T cells (Tregs) to inhibit anti-tumor immune responses. Additionally, LGMN^+^ macrophages modulate VEGF-A expression to facilitate angiogenesis. Collectively, these findings clarify the mechanism by which this specific cell subset facilitates GC progression, offering a new perspective on its malignant progression. Furthermore, this study positions LGMN^+^ macrophages as a potential immunotherapeutic target and a potential prognostic biomarker.

## Methods

### Data collection

We retrieved single-cell and bulk transcriptomic datasets, along with associated clinical data, from public databases. Specifically, single-cell RNA sequencing (scRNA-seq) data from patients with GC were obtained from the Gene Expression Omnibus (GEO, https://www.ncbi.nlm.nih.gov/geo/) GSE 183904 (14), comprising 10 matched non-tumor tissues and 26 tumor tissues. Bulk RNA-seq data and associated clinical information for patients with GC were obtained from public cohorts, including GSE15459 (15), GSE26253 (16), and The Cancer Genome Atlas database (TCGA, https://www.cancer.gov/ccg/research/genome-sequencing/tcga). We excluded samples lacking follow-up information in the subsequent analysis.

### Processing and annotation of scRNA-seq data

Cells were excluded from further analysis if they satisfied any of the following criteria: (1) Fewer than 500 detected genes; (2) more than 6,000 detected genes; or (3) a mitochondrial gene content exceeding 20%. Following this quality control, we focused our downstream analysis on the high-quality cells that met these criteria. The single-cell expression matrices were normalized and scaled using the LogNormalize method (scale factor = 10,000) in the Seurat R package. Batch effects among samples were further rectified using the Harmony package. For dimensionality reduction, the top 3,000 most variable genes were selected, and the top 50 principal components were utilized for downstream clustering and Uniform Manifold Approximation and Projection (UMAP) visualization.

Cell clustering was executed using a two-stage approach to ensure precise annotation. In the first stage, all cells were categorized and broadly annotated into major lineages based on canonical markers: epithelial cells (EPCAM and CDH1), T/NK cells (CD3D and CD3E), plasma B cells (CD79A and MZB1), endothelial cells (VWF and PLVAP), myeloid cells (CD14 and CD163), fibroblasts (DCN and COL1A1), B cells (CD19 and CD79A), mast cells (TPSAB1 and CPA3), and pericyte cells (RGS5). Clusters demonstrating co-expression of markers from incongruent lineages were identified and removed as potential doublets. In the second stage, cells from each major lineage were subsetted and independently re-clustered for enhanced resolution. Sub-clusters containing at least 200 cells were subsequently annotated using well-established, cell-type-specific marker genes or published gene signatures.

### Quantification of cell type infiltration using ssGSEA

The infiltration levels of various cell types in bulk tissue samples were quantified using single-sample gene set enrichment analysis (ssGSEA). Initially, cell-type-specific marker genes were identified from the annotated scRNA-seq data using the FindAllMarkers tool. For each cell type, the top 10 marker genes (ranked by log2 fold-change and detection rate) were aggregated into a signature gene set. These gene sets were employed to calculate per-sample infiltration scores for each corresponding cell type in the bulk RNA-seq cohorts from TCGA and GEO, utilizing the GSVA R package with the ssGSEA method. The obtained ssGSEA scores across all samples were normalized (z-scored) to ensure comparability in subsequent statistical analyses.

### Survival analysis

Kaplan–Meier survival curves were generated using the survfit function. The optimal cutoff for gene expression or cell type infiltration scores, established using the surv_cutpoint function from the survminer R package, was employed to categorize patients into high and low groups. These groups were compared using the two-sided log-rank test. Additionally, Spearman’s correlation analysis was conducted to assess the correlations between cell type infiltration proportions, with statistical significance established at a false discovery rate (FDR) of 0.05. The resulting correlations across different cell types within the GC cohort were visualized using the ggpubr R package.

### Functional enrichment and pathway analysis

Differentially expressed genes (DEGs) in the single-cell sequencing data were identified utilizing the FindMarkers function in Seurat. Gene ontology (GO) enrichment and Kyoto encyclopedia of genes and genomes (KEGG) pathway analyses were conducted using the clusterProfiler R package (17). Additionally, gene set enrichment analysis (GSEA) (18) was performed to ascertain if predefined biological pathways were enriched systematically. Gene sets for KEGG pathways were obtained from the Molecular Signatures Database (MSigDB). Enrichment scores for hallmark pathways were also calculated using gene set variation analysis (GSVA) to quantify the activity levels of specific biological programs within cell populations.

The enrichment scores for each individual cell were computed using the AddModuleScore function in Seurat. This allowed us to assess pathway activity at single-cell resolution. The gene sets utilized for this calculation were sourced from the Hallmark gene set collection in the MSigDB database (https://www.gsea-msigdb.org/gsea/msigdb/human/genesets.jsp?collection=H).

### Cell-cell communication analysis

To examine intercellular interactions among defined cell populations, we utilized NicheNet (19), a publicly available repository of curated ligand-receptor interactions, to deduce cell type-specific communication at the molecular level. A ligand or receptor was deemed “expressed” if it was detected in over 10% of the cells within a specific cluster. The ligand regulatory activity was plotted using the heatmap of ligand activity targets generated by NicheNet output. Activity scores ranged from 0 to 1.

### *In vivo* studies

LGMN^flox/flox^ C57BL/6 mice and *Lyz2-Cre* transgenic C57BL/6 mice were acquired from Shanghai Model Organisms Center, Inc. (Shanghai, China). *Lyz2-Cre* transgenic C57BL/6 mice express Cre recombinase under the control of the endogenous lysozyme 2 (Lyz2) promoter, which drives recombination in cells of the myeloid lineage, including macrophages, neutrophils, and some dendritic cell populations (20, 21). All mice were maintained under specific pathogen-free (SPF) conditions at Tianjin Medical University. To generate myeloid lineage-specific LGMN-knockout mice, LGMN^flox/flox^ mice were mated with *Lyz2-Cre* mice, resulting in LGMN^flox/flox^*; Lyz2-Cre* (abbreviated LGMN^mKO^ or mKO) offspring. Mouse genotypes were determined using polymerase chain reaction (PCR) analysis of genomic DNA extracted from tail biopsies. The PCR products were separated utilizing horizontal electrophoresis on a 1.5%–2.0% (w/v) agarose gel stained with ethidium bromide (or GelRed). Gel images were obtained under UV illumination, and genotypes were determined based on the presence and size of the expected DNA fragments.

YTN16, a mouse gastric cancer cell line established by Sachiyo Nomura and colleagues from a primary tumor in a C57BL/6 mouse (22), was used for *in vivo* experiments. This cell line is particularly valuable because immunocompetent C57BL/6 mice are generally resistant to transplantation of syngeneic tumor cells due to their strong immune system. YTN16 cells exhibit high expression of FGFR4 and possess the ability to metastasize and establish peritoneal dissemination, making them a clinically relevant model for studying gastric cancer progression and metastasis (23, 24). Therefore, we selected this cell line for our subcutaneous tumor model. In the subcutaneous tumor model, YTN16 cells (1 × 10^7^ cells per mouse) were subcutaneously injected into 6-week-old male LGMN^flox/flox^ (abbreviated as control or Ctrl) and LGMN^mKO^ mice (n = 4 per group). The mice were euthanized two weeks post-injection. Subcutaneous tumors were subsequently harvested, weighed, measured, and prepared for subsequent experiments. All experimental protocols involving mice were conducted in accordance with relevant ethical guidelines and were approved by the Ethics Committee of Tianjin First Central Hospital, affiliated with Tianjin Medical University.

### Immunofluorescence staining

Paraffin-embedded GC tissues and matched adjacent normal samples (n = 7 pairs) were procured from Tianjin First Central Hospital. All patients included in this study had not undergone preoperative radiotherapy or chemotherapy. All patients provided written informed consent for the utilization of their surgical materials for academic research. The collection of all specimens adhered to the regulations of the Institutional Review Board and the Medical Ethics Committee of Tianjin First Central Hospital. Dual-label immunofluorescence staining was performed utilizing Tyramide Signal Amplification (TSA) technology(G1259-50T, servicebio). Briefly, antigen retrieval was performed via microwave treatment, followed by blocking with 1% BSA. The first primary antibody was subsequently applied and incubated overnight. After adding the relevant horseradish peroxidase (HRP)-conjugated secondary antibody, the TSA reagent was administered and incubated for 10 min at room temperature in the dark. A second microwave treatment was conducted to remove the initial set of antibodies. The entire procedure was subsequently repeated for the next target. Nuclei were subsequently counterstained with DAPI. The samples were examined and imaged using a fluorescence microscope.

### Cell culture

The Cell Resource Center Affiliated with the Chinese Academy of Medical Sciences supplied the raw264.7 cell lines, YTN16 cell lines, and human umbilical vein endothelial cells (EA.hy926). Raw264.7, YTN16, and EA.hy926 cells were cultured in Dulbecco’s modified Eagle medium (DMEM) (Gibco BRL Life Technologies Inc., USA) with 10% fetal bovine serum (FBS) (life-ilab, AC03L055) and 1% penicillin-streptomycin (Hy-clone, CA, USA) at 37 °C in a 5% CO_2_ humidified cell incubator. We utilized RR-11A (25)(MCE, HY-112205), a synthetic enzyme inhibitor of legumain, to inhibit the enzymatic activity of LGMN in Raw264.7 cells.

### Isolation of bone marrow−derived macrophages

Male C57BL/6 mice aged 6–8 weeks were euthanized via cervical dislocation. Thereafter, the mice were immersed in 70% ethanol for 15 min. Skin and muscle tissues were carefully excised to preserve the integrity of bone marrow-derived progenitor cells. The femur and tibia were subsequently isolated from each leg. The bones were immersed in 70% ethanol and subsequently washed with phosphate-buffered saline on ice. The bone marrow was gradually extracted from each bone with DMEM medium. After collecting all bone marrow into a 50 mL tube, the cell suspension was filtered using a 200−mesh cell strainer and subsequently centrifuged at 300 ×g for 5 min at 4 °C. Following red blood cell lysis using an appropriate lysis buffer, the bone marrow pellet was resuspended in DMEM medium supplemented with 10% FBS, 1% penicillin−streptomycin−amphotericin B antibiotic−antimycotic solution, and 20 ng/mL Mouse M-CSF Recombinant Protein (315-02-10UG, Thermo fisher). The cells were subsequently seeded into 6−well plates and cultured for 6 days.

### *In vitro* tube formation assay

Conditioned medium was initially collected from Raw264.7 cells and BMDMs. Specifically, the treated macrophages were cultured in complete DMEM medium. After 24 h, the macrophage−conditioned medium (MCM) was harvested and centrifuged at 400 × g for 4 min. The supernatant was filtered through a 0.22 μm filter to remove debris. EA.hy926 cells were trypsinized and resuspended in the MCM. Matrigel was frozen on ice and subsequently dispensed into a 96−well plate at 50 μL per well, followed by polymerization at 37 °C for 30 min to create a gel. EA.hy926 cells (3 × 10^4^ cells per well) were inoculated onto the gel surface. After incubation at 37 °C under 5% CO_2_ for 2 h, three random fields per well were photographed using an inverted microscope.

### RT-qPCR and Western blot analysis

The total RNA was isolated from cells using TRIzol reagent (Invitrogen). Subsequently, we utilized complementary DNA (cDNA) reverse transcription kits to synthesize cDNA, and the mRNA expression levels were evaluated using qPCR in q7300. Gene expression was standardized to 18S mRNA levels and quantified utilizing the 2^ΔΔCt^ technique. The primer sequence for VEGF-A is as follows: F, GTCCGATTGAGACCCTGGTG, R, ACCGGGATTTCTTGCGCTTT (Sangon biotech). The primer sequences for the 18S gene are as follows. F, GGAAGGGCACCACCAGGAGT, R, TGCAGCCCCGGACATCTAAG (Sangon Biotech).

Western blot analysis was performed to evaluate the expression of target proteins. Total protein was extracted from cells or tissues utilizing RIPA-based lysis buffer (Cat#:R0010, Solarbio, China). Protein concentration was quantified using a bicinchoninic acid assay (Cat#:PC0020, Solarbio, China). Equal amounts of protein were separated using sodium dodecyl sulfate-polyacrylamide gel electrophoresis (SDS-PAGE Gel Preparation Kit, Cat#:AR0138, Boster, China; Electrophoresis buffer, Cat#:G2152-1L, Servicebio, China) and subsequently transferred onto polyvinylidene difluoride (PVDF) membranes. The membranes were blocked and subsequently incubated overnight at 4 °C with primary antibodies (LGMN, Cat#:93627S, diluted 1:1000, Cell Signaling Technology; VEGF-A, Cat#:19003-1-AP, diluted 1:1000, Proteintech; EIF5, Cat#: sc-135894, diluted 1:1000, Santa Cruz Biotechnology). After incubation with appropriate HRP-conjugated secondary antibodies (Goat anti-Rabbit IgG Secondary Antibody HRP conjugated, Cat#:L3012, diluted 1:10000, Signalway Antibody, China; Goat anti-Mouse IgG Secondary Antibody HRP conjugated, Cat#:L3032, diluted 1:10000, Signalway Antibody, China), protein bands were visualized using an enhanced chemiluminescence (ECL)(Cat#:E-IR-R308, Elabscience, China) detection method. All experiments were independently repeated at least thrice.

### Statistical analysis

The Wilcoxon rank-sum test was utilized for comparisons between the two groups. One-way analysis of variance (ANOVA) was utilized for analysis between three or more groups. R Studio and GraphPad Prism software were used for all statistical analyses. The threshold for significance was defined at *P* < 0.05.

## Results

### LGMN is upregulated in tumor-associated macrophages in GC

**Figure 1:**
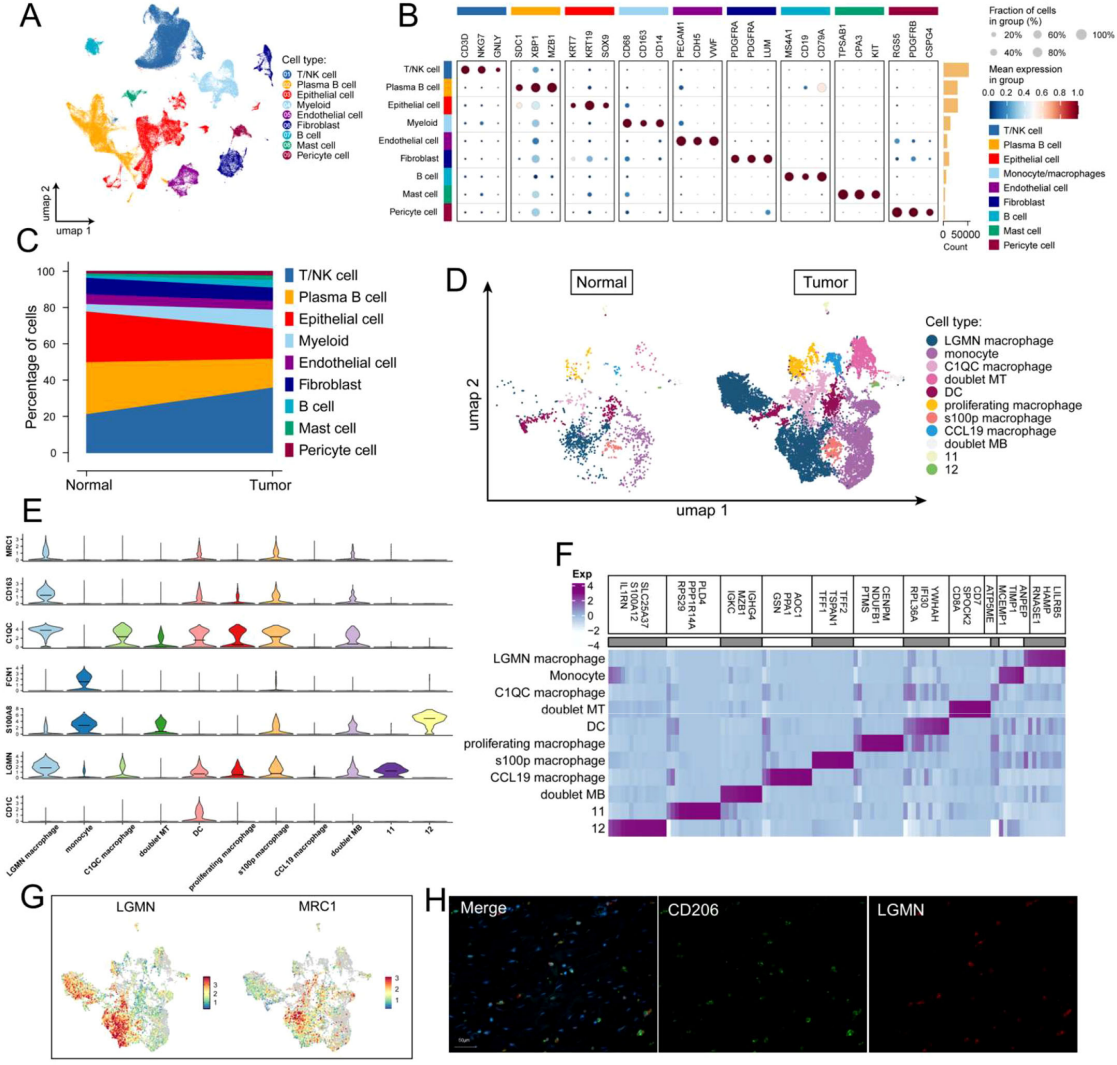
LGMN is upregulated in TAM of GC tissue. **(A)** UMAP visualization depicting 9 cell types from 157,084 single cells of 36 patients with GC. **(B)** Classical biomarkers for the annotated 9 cell types were identified. **(C)** Proportions of the identified 9 cell types in normal and tumor tissues. **(D)** UMAP plot illustrating 11 macrophage subsets in normal and tumor tissues. **(E)** Violin plots illustrating the expression levels of key marker genes for the 11 macrophage subsets. **(F)** The top 5 genes with the highest expression in each macrophage subset. **(G)** UMAP plot displaying the expression values of LGMN and MRC1 across macrophage subtypes. **(H)** Multiplex immunohistochemistry image of LGMN (red), CD206 (green), and DAPI (blue) staining in human GC tissues.

We conducted scRNA-seq analysis for 10 adjacent normal tissues and 26 GC samples, following rigorous quality control and filtration. We retained 150,913 high-quality cells that met our quality thresholds. Among these, 31,150 cells were derived from normal gastric mucosa, whereas 119,763 cells were sourced from GC tissues. To delineate the major cellular architecture, we conducted unsupervised clustering, which partitioned all cells into nine distinct clusters (**Figure 1A**). Each cluster was annotated utilizing canonical marker genes: T/NK cells (CD3D, NKG7, and GNLY), plasma B cells (XBP1 and MZB1), epithelial cells (KRT7, KRT19, and SOX9), myeloid cells (CD68, CD163, and CD14), endothelial cells (PECAM1, CDH5, and VWF), fibroblasts (PDGFRA and PDGFRB), B cells (MS4A1, CD19, and CD79A), mast cells (TPSAB1, CPA3, and KIT), and pericyte cells (RGS5) (**Figure 1B**). Although all cell types were present in normal and tumor tissues, their quantities varied significantly (**Figure 1C**). The proportion of monocytes/macrophages and T/NK cells was significantly increased in GC tissues relative to normal tissues.

TAMs affect the intrinsic characteristics of cancer cells and the TME. They can induce tumor cell proliferation, migration, and genomic instability and facilitate processes such as angiogenesis and lymphangiogenesis (26). We further subclustered myeloid cells and identified 11 distinct subsets (**Figure 1D**). We delineated specific immune cell subsets based on their marker expression: LGMN^+^ macrophages highly express LGMN; monocytes (express FCN1 and S100A9); dendritic cells (express CD1C and CD1E); proliferating macrophages (characterized by high MKi67); and S100p macrophages, and CCL19 macrophages exhibit high expression of S100p and CCL19, respectively. Additionally, we identified two populations of doublet cells that co-express markers of T cells or B cells (**Figure 1E**). **Figure 1F** highlights the top five highly expressed genes per subset. We found that LGMN^+^ macrophages constitute the highest proportion among TAMs, and their proportion was significantly increased in GC tissue compared with normal tissue. Moreover, we observed co-expression of LGMN and MRC1, which exhibited a positive correlation, indicating that LGMN^+^ macrophages possess an immunosuppressive phenotype similar to M2-polarized macrophages (**Figure 1G**). To further confirm this observation, we conducted immunofluorescence co-staining for LGMN and CD206 on GC tissues. The co-localization of these two markers offered substantial supporting evidence for this concept (**Figure 1H**).

### The infiltration of LGMN^+^ macrophages affects the survival prognosis of patients with GC

**Figure 2:**
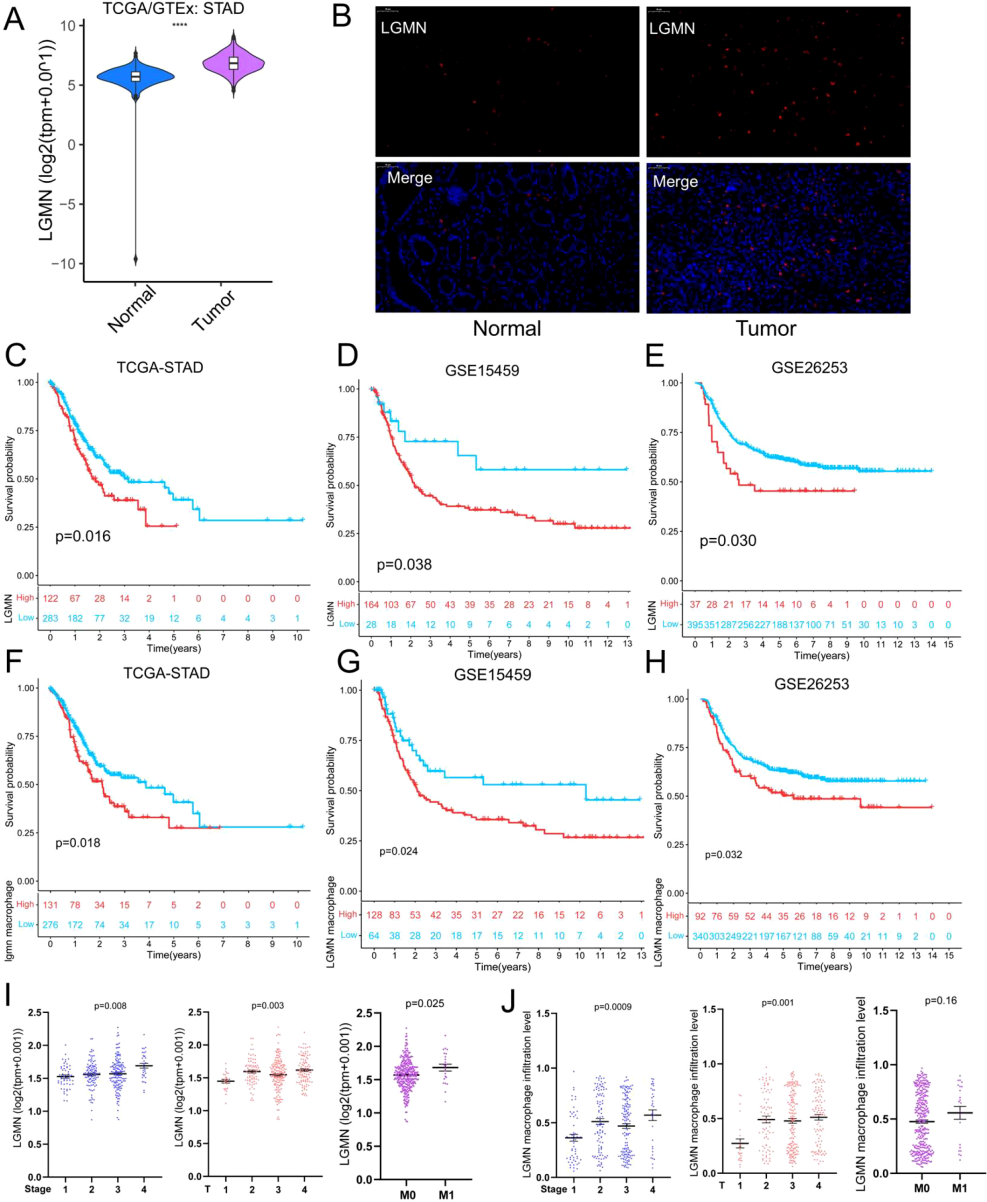
The infiltration of LGMN^+^ macrophages affects the survival prognosis of patients with GC. **(A)** Comparison of LGMN mRNA expression levels in GC and normal tissues. **(B)** Multiplex immunohistochemistry image of LGMN **(red)** and DAPI (blue) staining in human GC tissues and normal tissues. **(C–E)** Survival analysis demonstrates the association of LGMN mRNA levels with overall survival (OS) in patients with GC from TCGA and GSE15459 cohorts and with progression-free survival (PFS) in the GSE26253 cohort. **(F–H)** Survival analysis further revealed that high infiltration of LGMN^+^ macrophages was significantly associated with poor OS in TCGA and GSE15459 cohorts and with reduced PFS in the GSE26253 cohort. **(I)** Association between LGMN mRNA expression and tumor stage, tumor size and tumor metastasis. **(J)** Association between LGMN^+^ macrophages infiltration level and tumor stage, tumor size and tumor metastasis.

To clarify the function of LGMN in GC progression, we initially assessed its expression level in tumor versus normal tissues in the TCGA database. **Figure 2A** depicts that LGMN expression was significantly higher in GC tissues than in normal counterparts. To further investigate this, we performed LGMN immunofluorescence staining on paired tumor and adjacent normal tissue sections from patients with GC to assess its spatial distribution and clinical significance (**Figure 2B**). Consistently, LGMN^+^ macrophages were markedly more abundant in tumor tissues. We further examined the impact of LGMN expression levels and the infiltration level of LGMN^+^ macrophages on the prognosis of patients with GC (**Figures 2C–H**). Patients with high LGMN expression exhibited significantly poorer clinical outcomes compared to those with low expression. Similarly, patients with high infiltration of LGMN^+^ macrophages demonstrated poorer clinical outcomes. We then analyzed the correlation between LGMN^+^ macrophage and clinical parameters using public databases. LGMN mRNA expression value was positively correlated with tumor stage, tumor size, and metastasis (**Figure 2I**). In addition, LGMN+ macrophages infiltration level was also positively correlated with tumor stage and tumor size (**Figure 2J**). Our analyses confirmed the positive correlation between LGMN^+^ macrophages and these clinical indicators, implying that LGMN^+^ macrophages contribute to the progression of gastric cancer.

### LGMN^+^ macrophages are associated with hypoxia induction, angiogenesis, and immunosuppression

**Figure 3:**
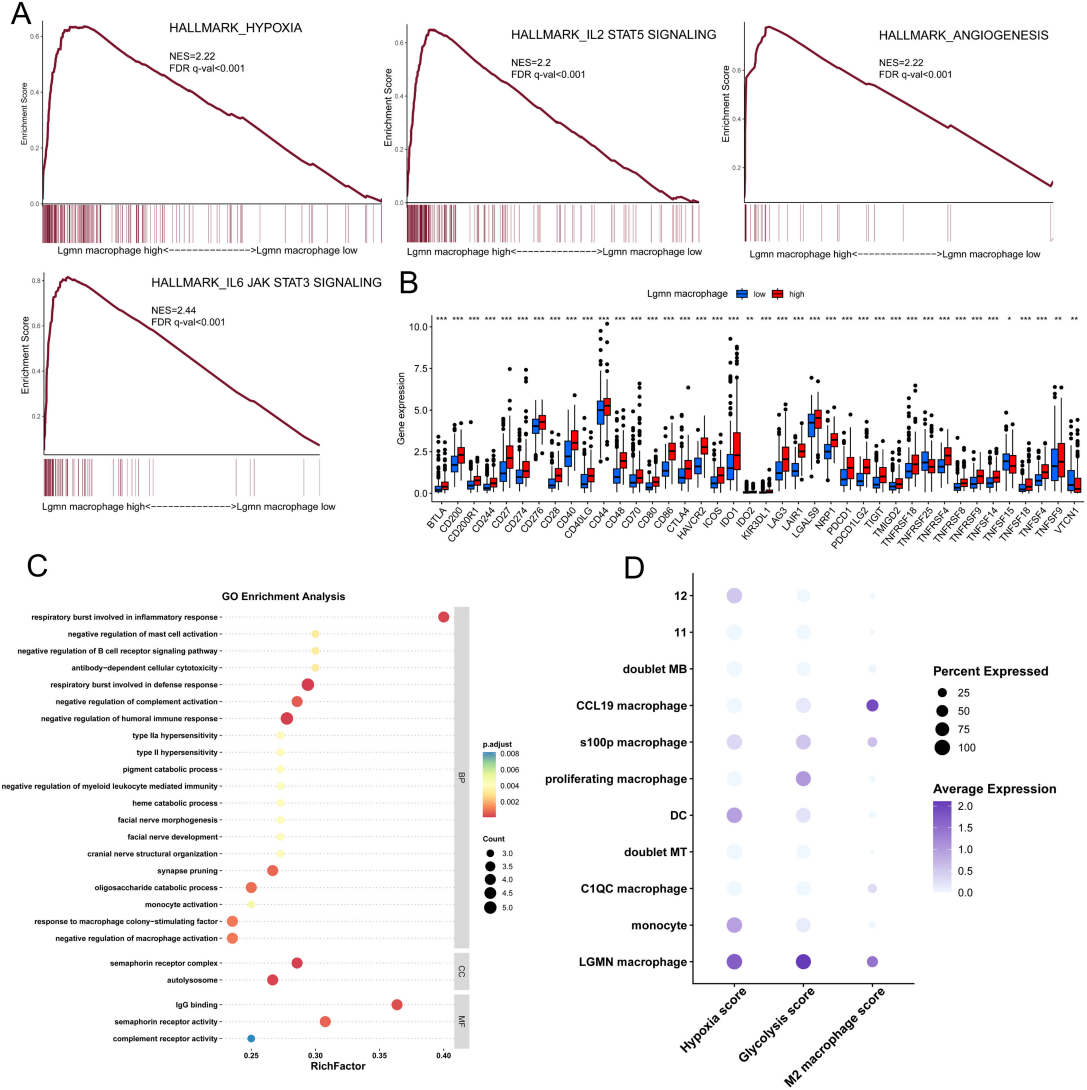
LGMN^+^ macrophages are associated with hypoxia, angiogenesis, and immunosuppression. **(A)** GSEA of hypoxia, angiogenesis, IL2-STAT5, and IL6-JAK-STAT3 signaling pathways between LGMN macrophage-high and LGMN macrophage-low groups. **(B)** Comparison of immune checkpoint expression levels between LGMN macrophage-high and LGMN macrophage-low groups. **(C)** GO analysis of the effector functions of LGMN^+^ macrophages. **(D)** Expression levels of hypoxia score, glycolysis score, and M2 macrophage markers across macrophage subtypes.

To examine the mechanism underlying the poor prognosis in patients with GC with LGMN^+^ macrophage infiltration, we conducted GSEA between LGMN^high^ macrophage groups and LGMN^low^ expression (**Figure 3A**). The LGMN^high^ macrophage group exhibited high enrichment of hypoxia-related signaling, indicating that the hypoxic TME is associated with LGMN upregulation in macrophages (27). Angiogenesis was highly enriched in this group, indicating a potential role of LGMN^+^ macrophages in tumor angiogenesis. Additionally, the LGMN^high^ macrophage group exhibited enrichment of IL6/JAK/STAT3 and IL2/JAK3/STAT5 signaling axes, which are crucial in tumor cell survival and T cell exhaustion (28–32). Simultaneously, patients with high infiltration of LGMN^+^ macrophages exhibited significantly higher expression of immune checkpoint molecules compared to those with low infiltration, indicating that this subset enhances the development of an immunosuppressive microenvironment (**Figure 3B**). These findings indicate that LGMN^+^ macrophages may facilitate GC progression by inhibiting anti-tumor immunity. We further examined the effector functions of LGMN^+^ macrophages using single-cell sequencing. Using the FindMarkers function in Seurat, we identified the differentially expressed genes (DEGs) of LGMN⁺ macrophages compared with other macrophage subsets, and performed Gene Ontology (GO) functional enrichment analysis using these DEGs. Compared with other macrophage subsets, GO analysis indicated that LGMN^+^ macrophages demonstrated enrichment in the negative regulation of humoral immune response, negative regulation of myeloid leukocyte-mediated immunity, negative regulation of macrophage activation, and negative regulation of complement activation (**Figure 3C**). Moreover, compared to other macrophage subsets, the enrichment of hypoxia, glycolysis, and M2 polarization was significantly elevated in LGMN^+^ macrophages (**Figure 3D**). These results collectively suggest that LGMN^+^ macrophages possess a broad immunosuppressive capacity, actively suppressing both adaptive (humoral) and innate (myeloid, complement) immune responses. The enrichment of these negative regulatory pathways indicates a potential self-reinforcing mechanism that maintains their immunosuppressive phenotype and prevents repolarization toward an anti-tumor state. These results collectively indicate that LGMN^+^ macrophages are associated with metabolic reprogramming, hypoxia, and immunosuppression.

### A correlation exists between LGMN^+^ macrophages and regulatory T cells

**Figure 4:**
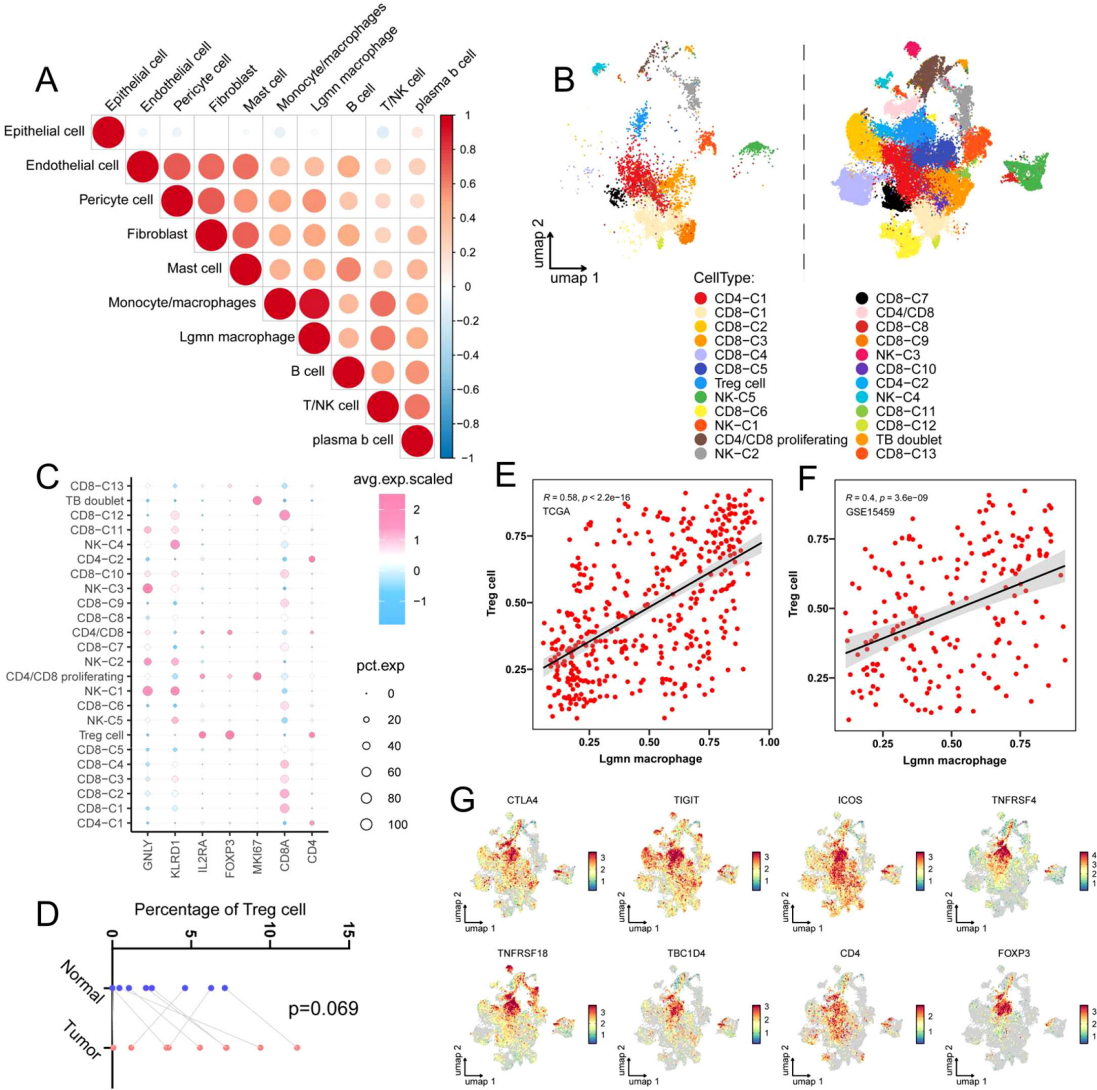
A correlation exists between LGMN^+^ macrophages and regulatory T cells. **(A)** Spearman correlation heatmap between LGMN^+^ macrophages and the other nine major cell types (red indicates positive correlation, blue indicates negative correlation). **(B)** T/NK cells in tumor and normal tissues were classified into 24 distinct subsets. **(C)** Dot plot illustrating the expression levels of key marker genes for each T cell subset. **(D)** The percentage of Treg cells in tumor tissues and the corresponding normal tissue. **(E, F)** Scatter plots demonstrating the correlation between LGMN^+^ macrophage infiltration and Treg cell abundance in two independent GC datasets, namely TCGA and GSE15459. **(G)** UMAP projection depicting the expression levels of CTLA4, TIGIT, ICOS, TNFRSF4, TNFRSF18, and TBC1D4 across T cell subsets.

TAMs act as an essential hub in the TME and engage in complex interactions with other cell types to affect tumor progression. To further examine the mechanism by which LGMN^+^ macrophages facilitate GC progression, we subsequently analyzed their correlations with other cell types. A strong correlation was observed between LGMN+ macrophages and endothelial cells and T/NK cells (**Figure 4A**). These findings suggest that LGMN^+^ macrophages may interact with both cell types. We therefore subdivided T cells into 24 subsets (**Figure 4B**), including regulatory T cells, CD4-C1, CD8-C1, CD8-C2, and CD8-C3, with key markers for each subset displayed in **Figure 4C**. Tregs, a specialized subset of T lymphocytes, are known for their immunosuppressive function and their role in promoting tumor progression (33). They play a significant role in the initiation and development of GC and serve as a prognostic biomarker (34). Depleting or inhibiting Tregs by targeted therapy has demonstrated promise in enhancing anti-cancer treatment responses and enhancing patient survival (34). Macrophages have been demonstrated to interact with Tregs through various mechanisms (33). We found that LGMN^+^ macrophages exhibited a strong positive correlation with Treg cells in TCGA and GSE15459 cohorts (**Figures 4E, F**), suggesting that LGMN^+^ macrophages may exert an immunosuppressive effect by recruiting, activating, or sustaining Tregs. Consistently, Tregs showed a trend toward higher abundance in tumor tissues compared to adjacent healthy tissues (**Figure 4D**). Furthermore, Treg cells exhibited high expression of immune checkpoint molecules, including CTLA4, TIGIT, and ICOS, and elevated levels of Treg activation markers, including TNFRSF18, TNFRSF4, and TBC1D4 (**Figure 4G**). Collectively, these findings indicate that Treg cells in GC tissue significantly suppress anti-tumor immunity.

### LGMN^+^ macrophages promote GC progression via immunosuppression

**Figure 5:**
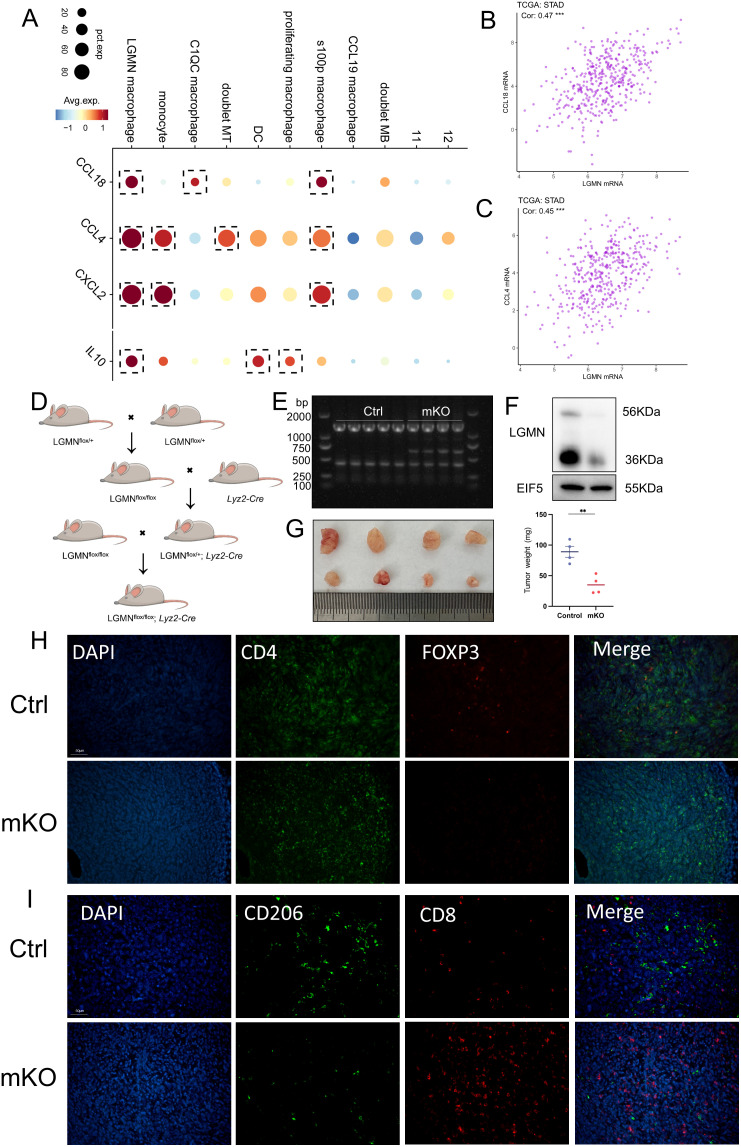
LGMN^+^ macrophages promote GC progression via immunosuppression. **(A)** Expression profiles of key immunomodulatory molecules across the 11 macrophage subsets. **(B, C)** The scatter plot illustrates the correlation between the expression of CCL18, CCL4 mRNA, and LGMN mRNA in the TCGA cohort. **(D)** Schematic diagram depicting the construction of the LGMN conditional knockout in macrophage mouse. **(E)** Genotyping identification of the LGMN^mko^ mice. **(F)** Western blot analysis of LGMN expression in BMDM (ctrl mice and LGMN^mko^ mice). **(G)** Subcutaneous tumor formation in mice and the corresponding bar graph of tumor weights. **(H)** Multiplex immunohistochemistry image of CD4 (green), FOXP3 (red), and DAPI (blue) staining in mouse subcutaneous tumor tissue. **(I)** Multiplex immunohistochemistry image of CD8 (red), CD206 (green), and DAPI (blue) staining in mouse subcutaneous tumor tissue.

We further investigated the mechanism of interaction between LGMN^+^ macrophages and Treg cells (**Figure 5A**). LGMN^+^ macrophages exhibited higher expression of chemokines, including CCL4, CCL18, and CXCL2, compared to other macrophage subsets. CCL18 can bind to the CCR8 receptor on Treg cells to recruit them, whereas CCL4 and CXCL12 can recruit Treg cells through the CCR5 and CXCR4 receptors, respectively (35, 36). Additionally, LGMN^+^ macrophages highly express IL-10, which regulates the suppressive function of Tregs (37) and is involved in Treg-mediated immune regulation and suppression (38). IL-10 can directly inhibit cytotoxic T lymphocyte (CTL) responses (39). Further analysis of the TCGA database revealed that the expression levels of CCL18 and CCL4 were positively correlated with LGMN expression. This suggests that LGMN^+^ macrophages may recruit Treg cells through the chemokines mentioned above (**Figures 5B, C**).

We subsequently performed *in vivo* experiments to confirm the role of LGMN^+^ macrophages. LGMN^flox/flox^*; Lyz2-Cre* (abbreviated LGMN^mKO^ or mKO) mice were generated (**Figure 5D**), and genotyping was conducted to confirm the knockout genotype, thereby ensuring the precision and reliability of our experiments (**Figures 5E, F**). Subcutaneous tumor models were then established by injecting YTN16 cells into these mice. The results demonstrated that the knockout of LGMN in macrophages significantly decreased tumor volume and weight (**Figure 5G**). Compared to control mice, LGMN^mKO^ mice exhibited increased infiltration of CD8^+^ T cells and a significant decrease in CD206, CD4, and Foxp3 expression (**Figures 5H, I**). These findings demonstrate that knockout of LGMN in macrophages was associated with reduced M2 polarization, reduces Treg recruitment, and enhanced anti-tumor immune responses in the tumor microenvironment. This suggests that LGMN^+^ macrophages may serve as a promising immunotherapeutic target.

### LGMN^+^ macrophages drive tumor progression by stimulating angiogenesis

**Figure 6:**
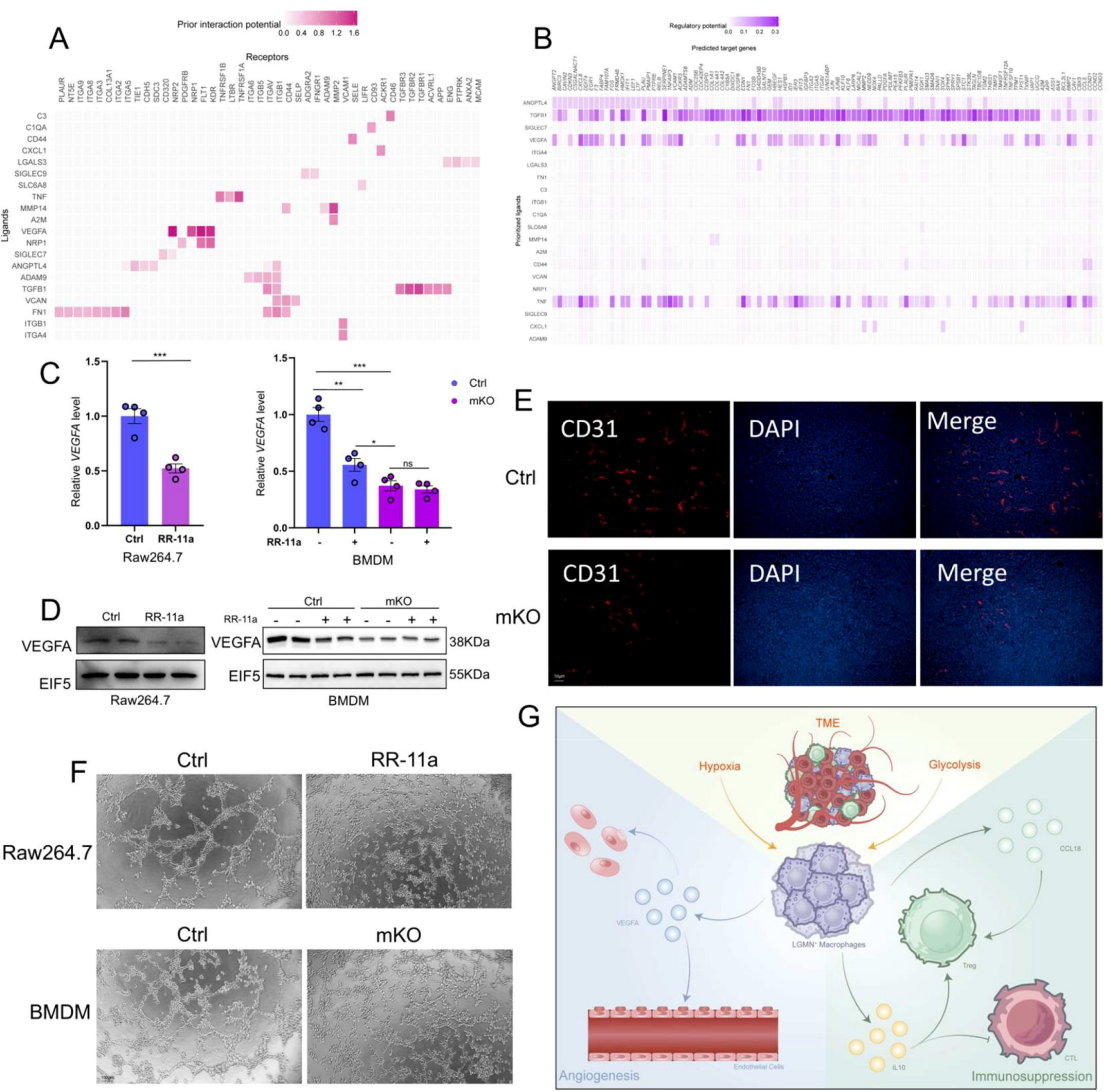
LGMN^+^ macrophages drive tumor progression by stimulating angiogenesis. **(A)** Network diagram of the predicted major ligand-receptor interactions between LGMN^+^ macrophages and endothelial cells in the GC. **(B)** The major predicted target genes of the ligands illustrated in Panel **(A)**, **(C)** VEGF-A mRNA levels evaluated by RT–qPCR in Raw264.7 cells treated with or without RR-11a, and in BMDMs from control and LGMN^mKO^ mice treated with or without RR-11a. **(D)** VEGF-A protein levels evaluated by Western blot in the same groups as in **(C)**. **(E)** Immunohistochemistry image of CD31 (red) and DAPI (blue) staining in mouse subcutaneous tumor tissue. **(F)** Analysis of the *in vitro* tube formation assay. (Top) Comparison of tube formation induced by conditioned medium from control Raw264.7 cells versus RR-11a-conditioned medium-treated Raw264.7 cells. (Bottom) Comparison of tube formation induced by conditioned medium from BMDM isolated from wild-type versus LGMN^mko^ mice. **(G)** Schematic diagram illustrating the mechanism by which LGMN^+^ macrophages facilitate GC progression. *P < 0.05, **P < 0.01, ***P < 0.001, ns: not significant (P ≥ 0.05).

As demonstrated in our earlier bioinformatic analysis (**Figure 4A**), we observed the infiltration levels of LGMN^+^ macrophages and endothelial cells were positively correlated. To further investigate the intercellular communication between these two cell types. We performed ligand-receptor interaction analysis using the NicheNet R package. LGMN^+^ macrophages and endothelial cells from normal tissues were designated as the reference sender and receiver populations, respectively. Multiple chemokine pairs were identified between the two subsets, including VEGF-A-KDR, VEGF-A-NRP2, VEGF-A-NRP1, VEGF-A-FLT1, TGFB1-ENG, TGFB1-TGFBR1, TGFB1-TGFBR2, TGFB1-TGFBR3, TGFB1-ACVRL1, and TGFB1-APP, highlighting the complexity of their communication (**Figure 6A**). Notably, we observed that the VEGF-A ligand exhibited the strongest interaction potential with its receptors KDR, NRP2, NRP1, and FLT1. Vascular endothelial growth factor (VEGF) is a potent survival factor for endothelial cells in physiological and tumor angiogenesis (40). KDR (VEGFR2) is the primary mediator of VEGF signaling in physiological and pathological conditions, representing the primary angiogenic axis that regulates endothelial cell proliferation, migration, survival, and vascular permeability (41). Notably, NRP2 can also enhance tumor-associated lymphangiogenesis, promoting cancer cell metastasis through lymphatic infiltration (42). We further analyzed the target genes regulated by these ligands (**Figure 6B**). VEGF-A was identified as co-expressed with genes including CXCL8, ACKR3, JUNB, ANGPT2, and EDN1. Among these, ANGPT2 (Ang-2) expression is a key factor in tumor angiogenesis and metastasis (43). Similarly, CXCL8 and EDN1 (Endothelin 1) also facilitate tumor growth by inducing angiogenesis (44, 45). To further examine whether LGMN facilitates angiogenesis by regulating VEGF-A, we treated Raw264.7 cells with the LGMN inhibitor RR-11a. The mRNA and protein levels of VEGF-A exhibited a significant decrease upon treatment. Similarly, treatment of control bone marrow-derived macrophages (BMDMs) with RR-11a significantly reduced VEGF-A expression at both mRNA and protein levels. Moreover, BMDMs from LGMN^mko^ mice exhibited significantly reduced VEGF-A expression compared to control mice, consistent with the inhibitor results (**Figures 6C, D**). To further confirm the angiogenic role of LGMN^+^ macrophages *in vivo*, we conducted immunofluorescence staining on subcutaneous gastric tumor tissues from LGMN^mko^ and control mice. The results revealed a significantly lower density of CD31^+^ vessels in the knockout group, suggesting impaired angiogenic function upon LGMN loss in macrophages (**Figure 6E**). At the functional level *in vitro*, we conducted a tube formation assay utilizing human umbilical vein endothelial cells (EA.hy926) exposed to the conditioned media of macrophages. The conditioned medium from RR-11a-treated Raw264.7 cells significantly attenuated vascular network development. Similarly, this consistent pattern was likewise observed in BMDMs (**Figure 6F**). Collectively, these findings suggest that LGMN^+^ macrophages facilitate tumor progression by regulating angiogenesis. **Figure 6G** presents a schematic overview of this mechanistic pathway.

## Discussion

TAMs are essential cellular components within the TME and are present at all stages of tumor progression. They can promote angiogenesis and enhance tumor cell invasion, motility, and intravasation. Additionally, TAMs exhibit immunosuppressive capabilities, preventing NK cells and T cells from targeting tumor cells during cancer progression and post-recovery after chemotherapy or immunotherapy (46). TAMs have conventionally been classified into M1 and M2 subtypes, with M1 typically viewed as an anti-tumor subtype and M2 perceived as facilitating tumor progression (47). However, current research has revealed an expanding diversity of TAM subsets with distinct functions (48–50). In our analysis of GC scRNA-seq data, we also identified more than two distinct macrophage subpopulations in patients with GC. Angiogenesis and immunosuppression during GC progression are major mechanisms contributing to the poor efficacy of chemotherapy, immunotherapy, and targeted therapy, presenting unresolved challenges for researchers. Consequently, identifying TAM subpopulations associated with angiogenesis and immunosuppression is essential for formulating effective therapeutic strategies for patients with GC. This study discovered a macrophage subset associated with poor prognosis in GC through single-cell RNA sequencing, termed LGMN^+^ macrophages. Notably, through immunofluorescence and subcutaneous tumor development assay, we further examined the mechanism by which LGMN^+^ macrophages facilitate GC progression. Our results indicate that LGMN^+^ macrophages stimulate angiogenesis and inhibit immune responses, thereby creating an immune microenvironment conducive to GC advancement.

We initially discovered that LGMN is predominantly expressed in TAMs and is significantly upregulated in tumor tissues. Subsequently, single-cell RNA sequencing and double immunofluorescence staining validated the co-expression of LGMN and CD206, demonstrating that LGMN^+^ macrophages exhibit characteristics of M2-polarized macrophages with tumor-promoting functions (**Figure 1F**). Previous studies reveal that LGMN functions as a prognostic marker and potential target in colorectal, breast, and ovarian cancers (51–53). Our study demonstrated that patients with GC with high LGMN expression have a poorer prognosis, and the infiltration level of LGMN^+^ macrophages is significantly correlated with adverse outcomes, highlighting its potential as a biomarker for monitoring and diagnosis. We further analyzed the characteristics of LGMN^+^ macrophages by integrating single-cell RNA sequencing and bulk RNA sequencing. IL2-STAT5 and IL6-JAK-STAT3 signaling pathways were significantly enriched in patients exhibiting high infiltration of LGMN^+^ macrophages. Moreover, patients with high infiltration exhibited significantly elevated expression of immune checkpoint molecules compared to those with low infiltration. Compared to other macrophage subsets, LGMN^+^ macrophages exhibited significant enrichment in immune-negative regulatory pathways. These findings collectively indicate the immunosuppressive nature of LGMN^+^ macrophages. Additionally, we validated this finding *in vivo*: knockout of LGMN in macrophages resulted in a significant downregulation of CD206 expression in tumors, confirming the immunosuppressive role of LGMN. Similarly, LGMN^+^ macrophages exhibited significant enrichment in hypoxia and glycolysis pathways, which may be associated with metabolic reprogramming in the TME.

TAMs undergo intrinsic modifications and facilitate tumor progression through interactions with other cell types within the TME (54). Given the complexity of cellular interactions within the TME, we utilized single-cell RNA sequencing to examine the interaction mechanisms between LGMN^+^ macrophages and other cell types. Our preliminary study revealed that LGMN^+^ macrophages exhibited the strongest correlations with T cells and NK cells. Subsequent analysis revealed a notably robust positive correlation with Tregs. Immunotherapy, especially immune checkpoint inhibitors (ICIs) and adoptive cell transfer therapy, has achieved significant clinical progress in cancer treatment (55, 56). Nonetheless, its efficacy in solid tumors is frequently limited by the immunosuppressive TME, wherein the infiltration of forkhead box protein P3-positive (FOXP3^+^) Tregs is a major obstacle (57–59). Treg cells, essential for maintaining immune homeostasis and preventing autoimmunity, constitute a significant barrier to anti-tumor immunity within the TME (35). Their intratumoral abundance correlates with poor prognosis in patients with GC (60) and significantly restricts the ability of CD4^+^ and CD8^+^ effector T (Teff) cells to eliminate tumors (59, 61). Therefore, precisely intervening in tumor-infiltrating Tregs without provoking systemic autoimmunity represents a core challenge for achieving effective therapy. Our study reveals that LGMN^+^ macrophages function as a key upstream hub modulating intratumoral Treg cell accumulation. We discovered that LGMN^+^ macrophages are highly correlated with Tregs in GC tissues and secrete key factors that drive Treg recruitment and differentiation. *In vivo* experiments provided causal evidence that LGMN^mko^ mice exhibited a marked reduction in the proportion of Tregs within subcutaneous gastric tumors, accompanied by inhibited tumor growth and enhanced CD8^+^ T cell infiltration. These findings indicate that LGMN^+^ macrophages are essential for maintaining a Treg-dependent immunosuppressive microenvironment. This discovery offers a novel perspective for overcoming immunotherapy resistance. As LGMN^+^ macrophages play a central role in Treg recruitment, they represent a rational upstream target for intervention. Given that macrophages can impair responses to T cell-based immunotherapies (62), targeting LGMN^+^ macrophages represents an indirect yet precise strategy. By reprogramming or eliminating this specific pro-tumorigenic macrophage subset, it may be possible to disrupt their continuous support for Tregs, thereby locally relieving immunosuppression, enhancing the efficacy of existing immunotherapies, and potentially avoiding the risk of systemic immune dysregulation associated with direct Treg targeting.

Furthermore, we observed a correlation between LGMN^+^ macrophages and vascular endothelial cells, indicating a potential role for LGMN^+^ macrophages in regulating tumor angiogenesis. The induction of neoangiogenesis is a hallmark of cancer (63). The abnormal structure and function of these tumor vessels contribute to malignancy by compromising blood perfusion (64, 65). Following extensive experimental confirmation of tumor-dependent angiogenesis, significant efforts have focused on developing anti-angiogenic therapeutic strategies. In 2004, the FDA’s approval of bevacizumab, the first anti-angiogenic drug, significantly prolonged PFS in patients with renal cell carcinoma when combined with chemotherapy. Because the VEGF/VEGFR signaling pathway is central to angiogenesis, most therapeutic strategies have targeted this axis, employing recombinant monoclonal antibodies or small-molecule tyrosine kinase inhibitors, constituting the mainstay of anti-angiogenic therapy (66). Despite considerable clinical progress, anti-angiogenic therapies continue to face limitations, including suboptimal efficacy and the development of drug resistance (63). This indicates the existence of underappreciated, VEGF-independent, or parallel pro-angiogenic mechanisms within the TME. Notably, our study identifies LGMN^+^ macrophages as a critical pro-angiogenic hub. Following LGMN knockout, the VEGF-A expression level in macrophages was decreased. Notably, single-cell RNA sequencing analysis indicated that the VEGF-A ligand exhibits the strongest interaction potential with its receptors KDR, NRP2, NRP1, and FLT1. Additionally, in LGMN^flox/flox^; *Lyz2-Cre* mice, tumor vascular density was significantly decreased. This evidence establishes LGMN^+^ macrophages as a key cellular source and amplifier of tumor angiogenesis. Notably, this study unifies angiogenesis and immunosuppression at the cellular level. First, tumor angiogenesis can induce immunosuppression and promote evasion of anti-tumor immunity. VEGF-A can directly or indirectly inhibit T cell development and function, induce T cell exhaustion through the upregulation of immune checkpoints, suppress dendritic cell maturation, modulate macrophage polarization, and increase the numbers of intratumoral Tregs and myeloid-derived suppressor cells (67). Preclinical studies indicate that the administration of anti-angiogenic agents favors the reversal of the immunosuppressive microenvironment into an immune-supportive one, hence augmenting the efficacy of vaccine-based immunotherapies (68). The anergic condition of tumor endothelial cells further substantiates that anti-angiogenic drugs enhance the efficacy of immunotherapy (67). Our study also has several limitations. First, the number of clinical samples in our study for the human IF validation is small. Second, although our findings confirm LGMN^+^ macrophages as a critical pro-angiogenic hub, the precise molecular mechanism by which LGMN regulates VEGF-A expression remains to be elucidated. Third, while our *in vivo* knockout model provides causal evidence that LGMN deficiency in macrophages reduces intratumoral Treg accumulation, the direct chemotactic effect of LGMN^+^ macrophages on Tregs remains to be validated. Fourth, we employed only one gastric cancer cell line (YTN16) for the *in vivo* experiments and did not include additional GC cell lines for further validation. Fifth, the human umbilical vein endothelial cell line EA.hy926 was employed, while the macrophage-conditioned medium was derived from mouse macrophages, thereby establishing a cross-species cell model system. Species differences between human endothelial cells and mouse macrophages may result in discrepancies in cytokine profiles, receptor recognition, and intracellular signaling pathways, which could compromise the reliability and translational potential of our findings. However, proteins from humans and mice share high homology, with highly similar structural and functional characteristics. Angiogenesis-related signaling pathways (including VEGF, Ang/Tie2, Notch, etc.) are highly conserved during mammalian evolution (66). Pro-angiogenic factors secreted by mouse macrophages can effectively activate the corresponding signaling axes in human endothelial cells, without interfering with the detection and evaluation of core phenotypes such as tube formation.

The identification of LGMN^+^ macrophages as a distinct pro-tumorigenic subset carries significant clinical implications. Our single-cell RNA sequencing analysis revealed that patients with higher infiltration of LGMN^+^ macrophages exhibited significantly poorer clinical outcomes, suggesting that this subset may serve as a valuable prognostic biomarker and a potential therapeutic target. Notably, this subset exhibited a distinctive dual functional gene signature encompassing immunosuppression and angiogenesis. Focusing on this specific cellular subset may yield a “two birds, one stone” effect: it could “starve” the tumor by cutting off its supply of pro-angiogenic factors and “activate” the immune system by relieving its suppression. This lays a theoretical foundation for developing novel therapies aimed at reprogramming the TME instead of merely blocking a single pathway.

## Data Availability

The original contributions presented in the study are included in the article/supplementary material. Further inquiries can be directed to the corresponding authors.
